# A radiostereometric and clinical long-term follow-up study of the surface replacement trapeziometacarpal joint prosthesis

**DOI:** 10.1186/s12891-021-03957-8

**Published:** 2021-02-05

**Authors:** Bart ten Brinke, Nina M. C. Mathijssen, Ian F. Blom, Lennard A. Koster, Gerald A. Kraan

**Affiliations:** 1 Department of Orthopaedic Surgery, Reinier de Graaf Groep, P.O. Box 5011, 2600 GA Delft, The Netherlands; 2 Department of Radiology, Reinier de Graaf Groep, P.O. Box 5011, 2600 GA Delft, The Netherlands; 3 Department of Orthopaedic Surgery, Leids Universitair Medisch Centrum, P.O. Box 5011, 2300 RC Leiden, The Netherlands

**Keywords:** Radiostereometry, Trapeziometacarpal joint, Trapeziometacarpal osteoarthritis, Trapeziometacarpal joint prosthesis, Migration

## Abstract

**Background:**

The aim of this study was to determine long-term survival and clinical outcomes of the surface replacement trapeziometacarpal joint prosthesis (SR™TMC) and to evaluate implant migration using radiostereometric analysis (RSA).

**Methods:**

In this clinical long-term follow-up study outcomes of ten patients who received the SR™TMC joint prosthesis were evaluated using DASH and Nelson scores, Visual Analogue Scale (VAS) of pain, and key pinch strength. RSA-radiographs were obtained direct postoperatively and 6 months, 1, 5 and 10 years postoperatively and were analyzed using model-based RSA software.

**Results:**

During follow-up, two early revisions took place. Mean pre-operative DASH and Nelson scores were 54 (SD 15) and 54 (SD 17), improved significantly after 6 months (DASH 25 (SD 20), Nelson 75 (SD 18)) and remained excellent during long-term follow-up in all patients with a stable implant. At final follow-up, clinical scores deteriorated clearly in two patients with a loose implant in situ.

**Conclusions:**

Long-term survival of the SR™TMC joint prosthesis is relatively poor. However, clinical outcomes improved significantly in the short-term and remained excellent in the long-term in those patients with a stable implant, but deteriorated clearly in case of loosening. The role of RSA in TMC joint arthroplasty is potentially valuable but needs to be further investigated. Several challenges of RSA in the TMC joint have been addressed by the authors and suggestions to optimize RSA-data are given.

**Trial registration:**

This study was registered in the Netherlands Trial Register (NL7126).

## Introduction

The two most widely used surgical procedures for the treatment of osteoarthritis (OA) of the trapeziometacarpal (TMC) joint are the trapeziectomy and TMC joint arthroplasty. In recently published research it is hypothesized that TMC joint arthroplasty is superior compared to trapeziectomy in terms of pain, strength, range of motion (ROM), satisfaction and recovery [1–5]. However, most studies present short-term follow-up and thus long-term data are of interest.

Previously, our group presented the 5-year results of a radiostereometric analysis (RSA) of the Surface Replacement (SR) TMC joint prosthesis (SR™TMC, Avanta®, San Diego, CA) [6]. This study showed a survival of eight out of ten prostheses with satisfying clinical outcomes.

The experience with RSA in the TMC joint is limited: two experimental and two small clinical studies have been published up to now [6–9]. These studies have learned us that RSA of the TMC joint is feasible with high precision for translations, but precision for rotation measurement is poor. Long-term RSA studies of the TMC joint have not been published before and thus long-term migration data of TMC joint prostheses are unknown.

The aim of the present study is to determine long-term survival and clinical outcomes of the SR™TMC joint prosthesis 10 years after placement and to evaluate the migration of the prosthesis during follow-up.

## Methods

### Design and participants

Ten consecutive patients (nine women) with osteoarthritis of the TMC joint received an SR™TMC joint prosthesis between June and October 2008 and were prospectively followed with a follow-up time of 10 years. Details of the original study are described in our previous paper. All participants of our previous study with the prosthesis in situ were invited to visit our clinic to undergo clinical and RSA examination and to complete patient reported outcome measures (PROMs).

### Clinical outcomes

To evaluate clinical outcomes, patients were asked to complete the Dutch version of the Disabilities of the Arm, Shoulder and Hand (DASH) and the Dutch translation of the Nelson Hospital Score [10, 11]. The DASH score decreases with functional improvement, whereas the Nelson Hospital score increases. Further, the Visual Analogue Scale (VAS) ranging from 0 to 100 was used to evaluate pain. Unlike in our short-term study, lateral pinch strength (key pinch) was measured at long-term follow-up (Mechanical Pinch Gauge, Sammons Preston, Bolingbrook, IL).

### Radiostereometric analysis

**Fig. 1:**
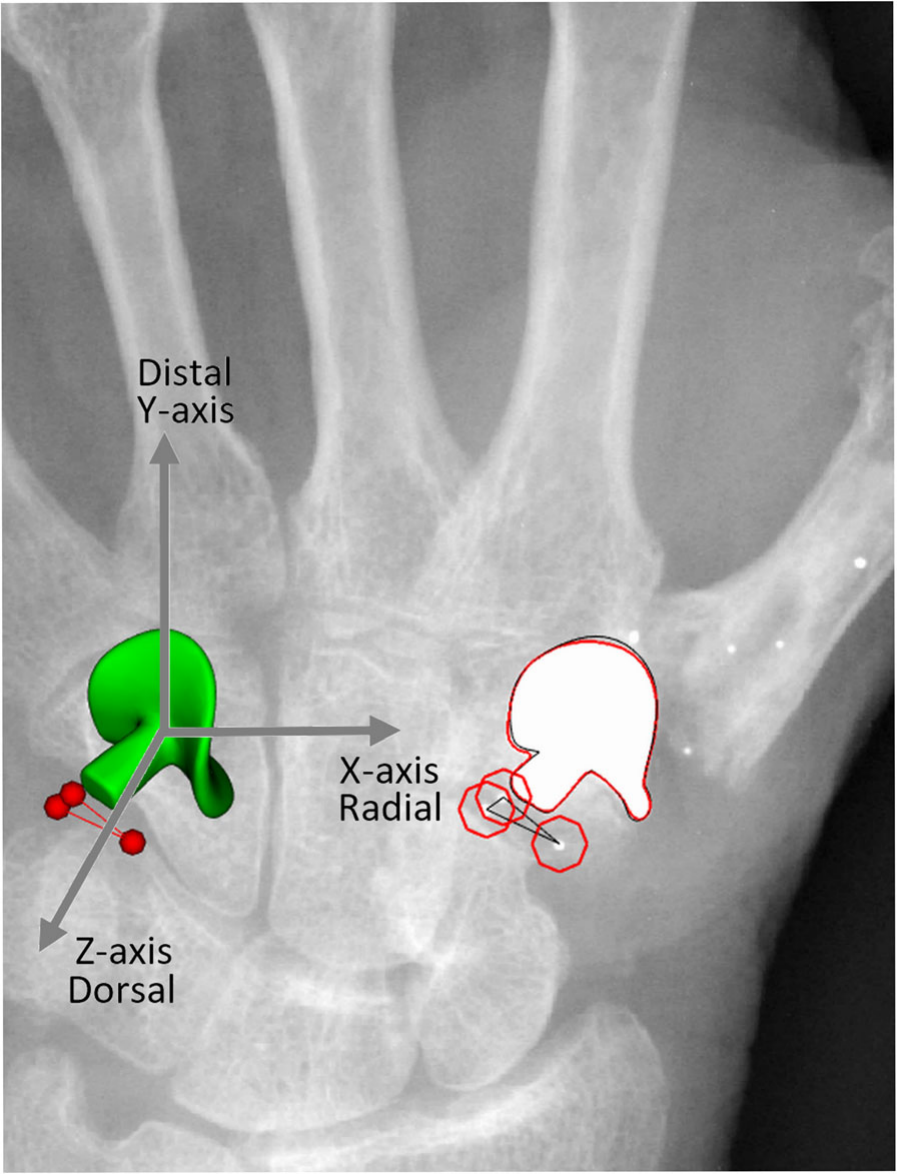
Detail of a model-based RSA scene analyzing migration of the SR^TM^TMC joint prosthesis. Three bone markers (red spheres) with a poor three-dimensional spatial distribution (CN 1016) resulting in poor rotational accuracy. The arrows indicate the three-dimensional coordinate system. Positive migration along the X-, Y-, and Z-axis indicates radial, distal and dorsal translation of the prosthesis with respect to the trapezial bone

RSA radiographs were taken using two synchronized roentgen tubes (DigitalDiagnost and the MobileDiagnost wDR (Philips, Best, The Netherlands)) positioned 1.2 m above the roentgen detector. The palm of the hand was placed on top of a Perspex calibration box (Medis, Leiden, the Netherlands). For each patient, all available RSA acquisitions were used to calculate migration with a model-based approach (Model-based RSA software version 4.2, RSA*core*, Leiden, The Netherlands). Migration is defined as translation (T; in mm) of the trapezial component with respect to the trapezium bone along the radial-ulnar (Tx), proximal-distal (Ty) and volar-dorsal (Tz) axis (Fig. 1).

Migration at all available follow-up moments was calculated with respect to the reference RSA acquisition taken direct postoperatively. In order to include as much data as possible, translations were calculated using the three-dimensional model of the implant as the reference object and the center of gravity of the bone markers as migrating object. As much as possible identical bone markers that could be detected in the RSA radiographs and meeting the International Organisation of Standardization (ISO) criterion for marker stability (Mean error (ME) < 0.35 mm) were used for translation measurements, even if the rigid body did not meet the ISO criterion for acceptable three-dimensional distribution (Condition Number (CN) < 150). The occluded markers model was applied to include bone markers not visible in particular RSA radiographs [12]. The calculated translations are multiplied with − 1 to express the results as translations of the implant with respect to the bone. Total translation (TT, mm) was calculated using the Pythagorean theorem (*√ (Tx*^*2*^ *+ Ty*^*2*^ *+ Tz*^*2*^*)*). Rotations were considered as inaccurate and not reported.

For all patients attending the 10-year follow-up, a double RSA examination was acquired to determine the precision of the technique. Precision was defined as 1.96 x standard deviation (SD) of ‘migration’ between two examinations taken at 10-year follow-up.

### Statistical analysis

Descriptive analysis was used to give an overview of survival rate. In order to investigate differences in DASH and Nelson scores a Wilcoxon signed-rank test was used. VAS pain scores, key pinch grip and migrations were described using descriptive analysis.

## Results

### Survival

Mean age at 10-year follow-up was 72 years (59–82). As reported in our previous paper two patients (patient 1 and 3) underwent a revision after respectively two and 3 years postoperatively because of persistent pain, without radiological signs of loosening. In one patient, progressive scaphotrapezial osteoarthritis was seen on conventional radiographs. In the other patient, the reason for persistent pain remained unclear. A trapeziectomy was performed in both patients. During revision surgery, both implants turned out to be well fixated. No additional prostheses were revised during follow-up. However, at 10-year follow-up two prostheses were clinically suspicious for loosening (patient 5 and 9), based on pain and loss of function. Single photon emission computed tomography (SPECT) showed increased uptake of technetium around the implant in both patients, indicating loosening. Conventional radiographs did not show any signs of loosening. Both patients were treated conservatively with a splint.

### Clinical outcomes

Of the ten patients enrolled in this study, eight patients with the prosthesis in situ completed 10-year follow-up. Mean pre-operative DASH and Nelson Hospital scores were 54 (SD 15) and 54 (SD 17). We previously found a statistically and clinically significant improvement in mean DASH and Nelson scores after 6 months (DASH 25 (SD 20), *p* = 0.04; Nelson 75 (SD 18), *p* = 0.02). Scores did not further improve or deteriorate between 6 months and 5 years (15 (SD 18), *p* = 0.4; 84 (SD 19), *p* = 0.4) nor between 6 months and 10 years (20 (SD 23), *p* = 1.0; 87 (SD 18), *p* = 0.3). VAS pain scores varied from 0 to 3 in rest and from 0 to 44 during activity on a 100-points scale in patients without clinical suspicion of loosening. As expected, DASH, Nelson and VAS pain scores worsened substantially in both patients with a suspicion of a loosening.

**Fig. 2:**
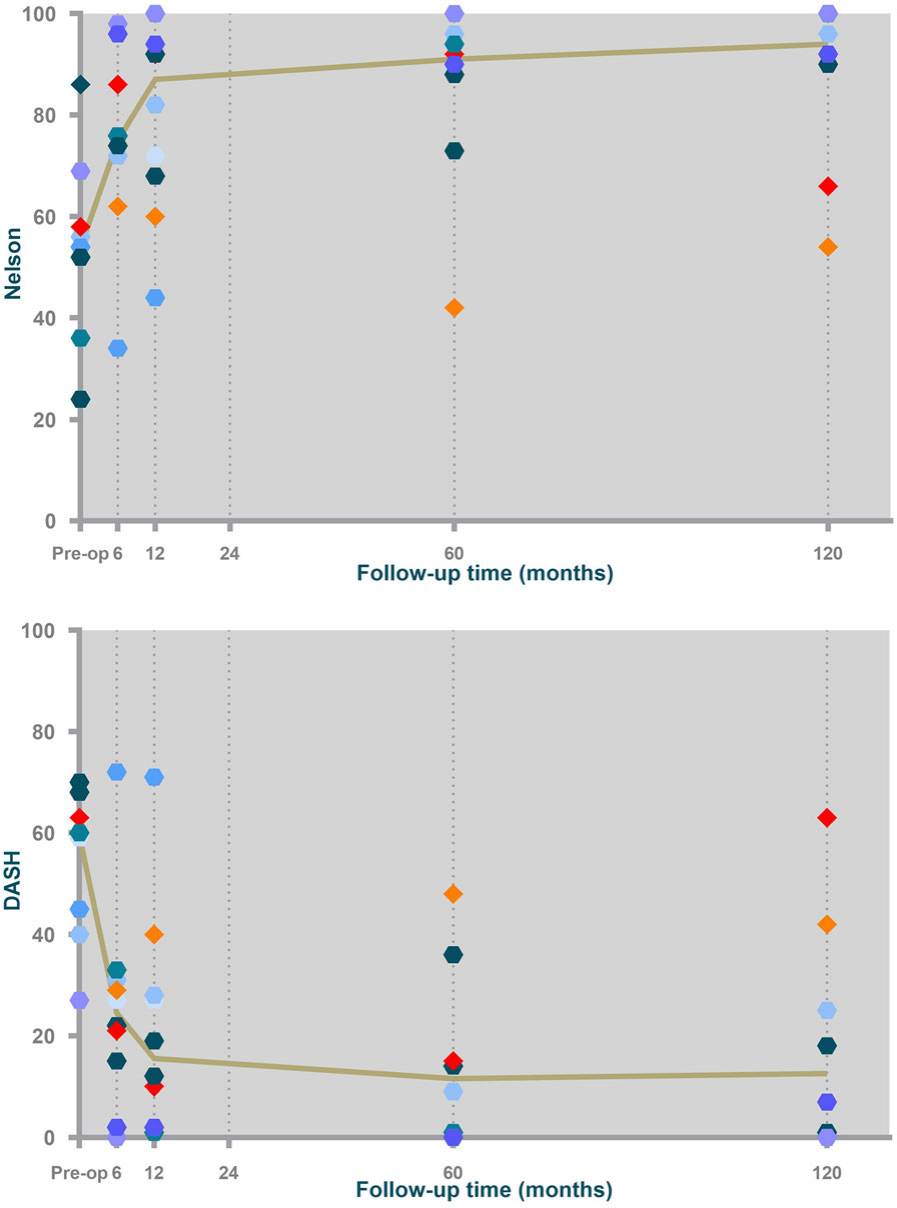
Median DASH and Nelson scores in ten patients with the SR^TM^TMC joint prosthesis. Median DASH and Nelson scores are indicated by the curve. Single patients are expressed as markers. Patients with a loose implant are expressed as orange (patient 5) and red (patient 9) diamonds

Key pinch strength was remarkable high in our study population. Clinical scores are summarized in Table 1 and Fig. 2.

**Table 1:** Patient characteristics and clinical results 10 years after implantation of the SR™TMC joint prosthesis

Patient ID	Age at surgery	Sex	Side	VAS pain (rest)	VAS Pain (activity)	Key pinch strength (kg)
^a^1	64	F	L	.	.	.
2	67	F	R	3	20	6
^a^3	70	F	R	.	.	.
4	59	M	R	0	0	13
^b^5	56	F	L	75	74	3
6	61	F	R	0	0	9
7	49	F	R	2	44	9
8	59	F	L	0	0	8
^b^9	58	F	L	45	66	4
10	72	F	R	1	1	3

^a^Patient who underwent revision surgery

^b^Patients with loose implant at 10-year follow-up

### RSA results

Of the eight patients who completed 10-year follow-up, RSA radiographs were taken in seven patients. One patient received a ‘small’ sized implant, of which no Computer-aided design (CAD) model was available in the software and thus RSA radiographs were not acquired. Double examinations could be used in six patients to determine precision of RSA. One patient had not enough markers visible in the double examination. Precision values of translations along the x-, y- and z-axis are given in Table 2.

**Table 2:** Precision measurement using double examinations in six patients with the SR™TMC joint prosthesis in situ

	Tx	Ty	Tz	Total translation
Min	−0.09	−0.01	−0.15	0.04
Max	0.08	0.13	0.12	0.19
Median	0.01	0.02	0.01	0.11
Mean	0.01	0.03	0.00	0.11
SD	0.06	0.05	0.10	0.06
Upper 95% CI	0.14	0.13	0.20	0.22

**Fig. 3:**
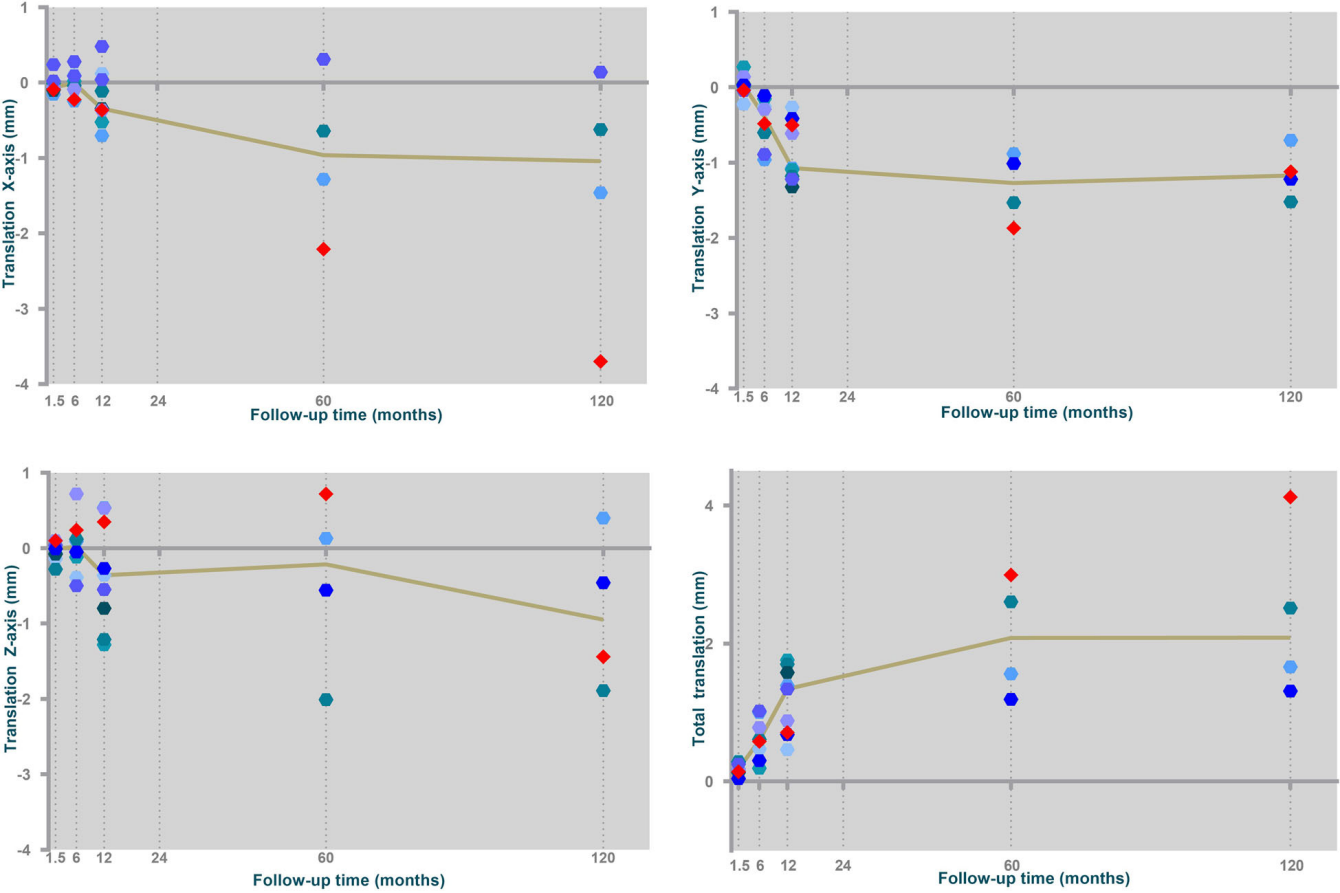
Median translations along the X-, Y- and Z- axis and Total Translation of the trapezial component of the SR^TM^TMC joint prosthesis with respect to the trapezial bone during 10 years of follow-up. Median translation values are indicated by the curve. Single patients are expressed as markers. Patients with a loose implants (patient 5 and 9) are expressed as red diamonds

In four patients we were able to calculate translations of the implants up to 10 years of follow-up. In the other patients, translations were calculated up to the last follow-up moment with analyzable RSA radiographs, but not up to 10 years postoperatively because of revision of the implant (*n* = 2), lack of visible bone markers (*n* = 1) or unstable bone markers (ME > 0.35, *n* = 2). An overview of all translations is shown in Fig. 3.

A stable migration pattern was seen in three implants. In one of the patients clinically suspicious for implant loosening (patient 9) RSA confirmed increased migration of the implant (Fig. 3), despite negative conventional radiographs. In the second patient with a clinically suspicious loose implant (patient 5) RSA radiographs could not be analyzed because of instable markers.

## Discussion

In this first long-term RSA-study of the TMC joint we present 10-year survival rate, clinical outcomes and migration of the SR™TMC-joint prosthesis. After 10 years of follow-up, two out of ten prostheses were revised and two additional loose implants were found at the 10-year follow-up moment. Long-term survival is worse compared to recently published long-term results of the Roseland®, ARPE®, Ivory® and Rubis II prostheses, varying from 85 to 95% [13–18]. This is mainly explained by two early revisions. Migration analysis of these implants showed a stable fixation of the trapezium component in the first 12 months postoperatively and both implants turned out to be well fixated during revision surgery. Therefore, both revisions were not considered as loose implants. No additional implants were revised during further follow-up. However, at 10-year follow-up, two patients (patient 5 and 9) had clinical signs of loosening including pain and loss of function. Clinical suspicion was supported in patient 9 by RSA as we found the implant migrating substantially, while conventional radiographs did not show any sign of loosening. Loosening could not be confirmed by RSA in patient 5 since analysis of the RSA radiographs was inaccurate as a result of unstable markers.

Six patients were highly satisfied with high DASH and Nelson scores and low VAS pain scores. Especially key pinch grip was high in these patients and comparable with pinch grip in the normal population corrected for sex and age. These satisfying results are comparable with previously published long-term results [13–18].

Several studies have been published comparing total joint arthroplasty with trapeziectomy, but only reporting short-term results [1–5]. Jager et al. described higher satisfaction, mobility, strength, pain reduction and functional scores in favor of the MAIA® total joint prosthesis [1]. Robles-Molina et al. reported similar pain relief and functional improvement, but superior pinch strength and range of motion in the ARPE group [2]. Besides a significantly better ROM, pinch strength, DASH, pain relief and satisfaction, Cebrian-Gomez et al. described a faster return to work in the Ivory prosthesis group [3]. Unlike the results of Cebrian-Gomez et al., Thorkildsen and Røkkum did not find any significant difference in DASH scores between TMC joint arthroplasty and trapeziectomy but did find better motion and strength in the prosthesis group [4].

On the other hand, most studies show higher complication and revision rates in total joint arthroplasty. Taking this into consideration, together with the assumed higher costs of TMC joint arthroplasty in comparison with trapeziectomy, the optimal surgical treatment for TMC joint OA remains a topic of debate. In our opinion further research should be done to investigate which individual patients do actually have benefit from the described advantages of total joint arthroplasty and which do not.

Worth noticing is that all controlled trials comparing trapeziectomy and total joint arthroplasty have investigated ball-and-socket design implants and not saddle-shaped SR implants as used in this study. The SR TMC joint prosthesis has been developed to preserve normal anatomy and kinematics of the thumb, striving for better survival [19]. Although controlled trials comparing the two implants have not been performed, short-term survival of the SR implant appeared to be inferior to ball-and-socket implants whereafter the prosthesis was withdrawn from the market [20]. Apart from our study, no long-term results of the SR TMC joint prosthesis are available to compare with long-term results of ball-and-socket designs. Given the lack of long-term survival data and the absence of controlled studies, no firm conclusions can be drawn about superiority of one of both implants in the long term.

Concerning the role of RSA in TMC joint arthroplasty scientific support is limited. RSA studies analyzing TMC joint prostheses are sparse and patient cohorts are small. Furthermore, the technique faces some significant challenges that have to do with the small size of the joint. As in previously published studies, it was not possible to calculate rotations of the implant in our study. The main reason for this is the lack of stable (ME < 0.35), sufficient (*N* > 2) and well three-dimensional spread (CN < 150) markers. Using marker rigid bodies containing unstable and poor spread markers results in large and incorrect rotations. Simply ignoring these rotations is not the right strategy since rotations do affect translation calculation. An incorrect rotational alignment of the reference model can lead to incorrect rotations and hence inaccurate translation measurement. This effect of rotation on translation is explained by Beardsley et al. and Van Hamersveld et al. [21, 22] Therefore, striving for a well three-dimensional spread and stable rigid body as a reference for migration calculation is still important, even if rotations are left out of consideration. However, the small size of the surrounding bone makes it difficult to ensure this in the TMC joint. In cases were RSA data cannot be analyzed due to said reasons the alternative strategy of *reversed migration* calculation as applied in this study may be a better option for two reasons.

First, when the implant is used as the reference model, RSA scenes with only two bone markers can be used for translation calculation. The reference model can be used to correct for the different positions of the TMC joint with respect to the calibration cage in different RSA acquisitions. With only two markers in the reference model, this cannot be accurately done since two markers create a line around which the implant model can be rotated 360 degrees, resulting in potentially inaccurate migrations.

Secondly, for marker rigid bodies with a CN > 150 similar issue arises. The higher the CN, the more the markers in the rigid body are aligned in a column like fashion, resulting in similar rotational inaccuracies as described above (Fig. 1). The strategy of reversed migration calculation will not solve all marker-related problems but does allow for more data to be analyzed. Furthermore, an improvement can be expected in translation measurements when marker rigid bodies have a high CN.

To avoid the problem of invisible or unstable markers, a CT-based method has been developed with comparable accuracy and precision to that of RSA of the hip [23]. More recently, Broden et al. described the use of a CT-based method to calculate migration of shoulder implants [24]. In an experimental setting, accuracy and precision were comparable to that of RSA with similar effective doses. Given the mentioned marker-related problems, accuracy and precision of CT-based migration calculation is worth to be investigated in the TMC joint.

Considering the proved predictive value of early migration in hip- and knee arthroplasty for future loosening, RSA plays an important role in the introduction and surveillance of orthopedic implants [25–27]. However, the relation between early migration and future loosening has not yet been proved in other joints than the hip and knee. If existent, this relation will be difficult to demonstrate in TMC joint arthroplasty because of small patient numbers. Thus, the question arises ‘What to do with RSA in the TMC joint?’

An important principle in total joint replacement is to strive for the best possible fixation of implants into the surrounding bone. Although the predictive value of early migration of TMC joint implants is unclear, RSA remains the most accurate method available to assess implant migration and fixation. Comparing different implant designs, RSA may play an important role in the early distinction of good and bad performing implants preventing implants with suboptimal fixation coming into the market.

A not so often discussed feature of RSA is the use of the technique as a diagnostic tool in individual cases. In daily practice, confirming clinical suspicion of loosening may be challenging, expensive and time consuming. Generally, the first step in the diagnostic algorithm of implant failure is taking conventional radiographs. However, the value of conventional radiographs in diagnosing loosening is limited [28]. Magnetic Resonance Imaging (MRI) and Computed Tomography (CT) scans to assess implant loosening are more expensive and generally difficult to read because of metal artefacts [29]. Additional bone scintigraphy and SPECT may be helpful but are expensive and time consuming.

In our study we found two patients with clinical symptoms of loosening. Conventional radiographs did not show signs of loosening, while RSA enabled us to easily confirm implant migration in one of both patients. Although the numbers in this study are too small to come to conclusions, future research comparing diagnostic accuracy and cost-effectiveness of RSA versus other diagnostic modalities in the detection of implant loosening could be interesting. The diagnostic value of RSA could be investigated in patients who are already involved in RSA studies and could undergo both RSA examinations and other diagnostic tests in case of loosening.. Alternatively, tantalum beads could be implanted in new patient cohorts undergoing total joint arthroplasty. After obtaining two postoperative RSA radiographs as reference and to demonstrate stabilization of implants, RSA radiographs could be repeated in case of clinically suspicion of loosening during follow-up.

## Conclusion

Long-term survival of the SR™TMC joint prosthesis is relatively poor. Clinical outcomes improved significantly in the short-term and remained excellent in the long-term in those patients with a stable implant, but deteriorated clearly in case of loosening. The role of RSA in TMC joint arthroplasty is potentially valuable but needs to be further investigated.

## Data Availability

The datasets used and analysed during the current study are available from the corresponding author on reasonable request.

## Abbreviations

CAD: Computer-Aided Design
CN: Condition number
DASH: Disability of the Arm, Shoulder and Hand
ISO: International Organisation of Standardization
ME: Mean Error
OA: Osteoarthritis
PROM: Patient Reported Outcome Measures
ROM: Range Of Motion
RSA: Radiostereometric Analysis
SD: Standard Deviation
SPECT: Single Photon Emission Computed Tomography
SR: Surface Replacement
T: Translation
TT: Total Translation
TMC: Trapeziometacarpal
VAS: Visual Analogue Scale
